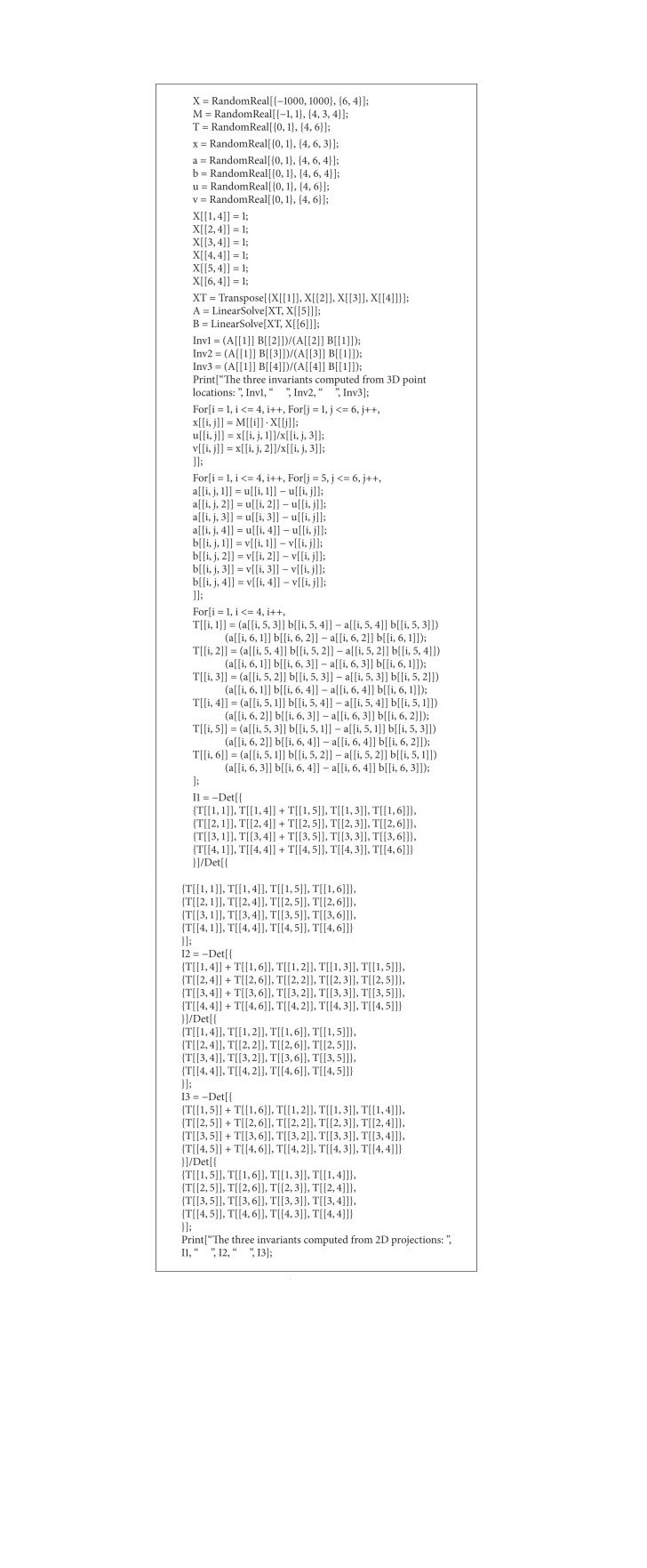# A Linear Method to Derive 3D Projective Invariants from 4 Uncalibrated Images

**DOI:** 10.1155/2014/109318

**Published:** 2014-01-29

**Authors:** YuanBin Wang, XingWei Wang, Bin Zhang, Ying Wang

**Affiliations:** ^1^College of Information Science and Engineering, Northeastern University, Shenyang 110819, China; ^2^Department of Computer Science, Worcester Polytechnic Institute, Worcester, MA 01609, USA

## Abstract

A well-known method proposed by Quan to compute projective invariants of 3D points uses six points in three 2D images. The method is nonlinear and complicated. It usually produces three possible solutions. It is noted previously that the problem can be solved directly and linearly using six points in five images. This paper presents a method to compute projective invariants of 3D points from four uncalibrated images directly. For a set of six 3D points in general position, we choose four of them as the reference basis and represent the other two points under this basis. It is known that the cross ratios of the coefficients of these representations are projective invariant. After a series of linear transformations, a system of four bilinear equations in the three unknown projective invariants is derived. Systems of nonlinear multivariable equations are usually hard to solve. We show that this form of equations can be solved linearly and uniquely. This finding is remarkable. It means that the natural configuration of the projective reconstruction problem might be six points and four images. The solutions are given in explicit formulas.

## 1. Introduction

The recovery of the geometric structure of 3D points from 2D images is fundamental in computer vision. After decades of research, most of the mathematical aspect of this problem is well understood. It is proved that the geometric information of a 3D point configuration cannot be recovered from a single image, unless the configuration is further constrained [1]. When two or more images are available, the 3D structure of a scene can be recovered up to an unknown projective transformation. The projective reconstruction of camera parameters and 3D scene structure from multiple uncalibrated views is also called projective structure and motion [1–5].

A camera is a device that transforms properties of a 3D scene onto an image plane. A pinhole camera model is used to represent the linear projection from 3D space onto each image plane. In this paper, 3D world points are represented by homogeneous 4-vector **X**_*i*_ = (*x*_*i*_,*y*_*i*_,*z*_*i*_,1)^*T*^. The projection of the *i*th 3D point is represented by a homogeneous 3-vector **x**_*i*_ = (*u*_*i*_,*v*_*i*_,1)^*T*^. The relationships among the 3D points **X**_*i*_ and their 2D projections are

(1) $$\begin{array}{c} k_{i}^{j}x_{i}^{j}=P^{j}X_{i},\;i=1,\ldots ,n,\;\;j=1,\ldots ,m, \end{array}$$

where **P** ^*j*^ is the projection matrix (which is 3 × 4 and is also called the camera matrix) of the *j*th camera, *k*_*i*_^*j*^ is a nonzero scale factor called projective depth, and **x**_*i*_^*j*^ = (*u*_*i*_^*j*^, *v*_*i*_^*j*^, 1)^*T*^ is the *j*th projection of the *i*th 3D point. Suppose that *m* perspective images of a set of *n* 3D points are given. The structure and motion problem is to recover the 3D point locations and camera locations from the image measurements. When the cameras are uncalibrated and no additional geometric information of the point set is available, the reconstruction is determined only up to an unknown projective transformation. For any 3D projective transformation matrix *H*, *P*_*i*_ *H* ^−1^ and *HX*_*i*_ produce an equally valid reconstruction.

Existing methods for projective reconstruction are usually indirect. They rely on a priori estimation of some tensors of multiple images of the scene to estimate the 3D point structure. A second-order tensor usually called the fundamental matrix captures the geometry between two views of a 3D scene. A third-order tensor usually called trifocal tensor captures the geometry among three views of a 3D scene. When these tensors of multiple views of a scene are known, there are many algorithms to recover the 3D geometric structure of the scene from them [6–17].

We can also compute 3D projective invariants of a point set from its 2D images directly. In the famous paper [9], Quan proposed a method to compute 3D projective invariants of six 3D points from three uncalibrated images. However, the method proposed by Quan is rather complicated and hard to use in real applications.

This paper presents a fast linear method for computing projective invariants of six 3D points from four 2D view images. A 3D point structure can be configured by first choosing four reference points as a basis and then representing the other two points under this basis. The cross ratios of the coordinates of the other two points under this basis are projective invariant. A system of four bilinear equations in three unknowns is derived first. Traditional methods to solve nonlinear multivariable equations are very complicated. The main contribution of this paper is that we will show that this system of equations can be easily transformed into some linear equations. This finding is remarkable. It means that the natural configuration of the projective reconstruction problem is six points and four images. The projective invariants are given in explicit formulas.

## 2. Related Works

We review a few related works in this section. The most famous of which is the work of Quan [9].

In [1], Faugeras studied projective reconstruction using five reference points called standard basis whose homogeneous coordinates are

(2) $$\begin{array}{c} E_{1}=\left(\begin{array}{c} 1\\ 0\\ 0\\ 0 \end{array}\right),\;\;E_{2}=\left(\begin{array}{c} 0\\ 1\\ 0\\ 0 \end{array}\right),\\ E_{3}=\left(\begin{array}{c} 0\\ 0\\ 1\\ 0 \end{array}\right),\;\;E_{4}=\left(\begin{array}{c} 0\\ 0\\ 0\\ 1 \end{array}\right),\;\;E_{5}=\left(\begin{array}{c} 1\\ 1\\ 1\\ 1 \end{array}\right). \end{array}$$

Suppose that the 3D points *E*_1_, *E*_2_, *E*_3_, and *E*_4_ are transformed by each camera into 2D image points

(3) $$\begin{array}{c} e_{1}^{j}=\left(\begin{array}{c} 1\\ 0\\ 0 \end{array}\right),\;\;e_{2}^{j}=\left(\begin{array}{c} 0\\ 1\\ 0 \end{array}\right),\\ e_{3}^{j}=\left(\begin{array}{c} 0\\ 0\\ 1 \end{array}\right),\;\;e_{4}^{j}=\left(\begin{array}{c} 1\\ 1\\ 1 \end{array}\right). \end{array}$$

They form a projective basis for the *j*th image plane. Then the reduced camera matrix looks like

(4) $$\begin{array}{c} P^{j}=\left(\begin{array}{cccc} a^{j} & 0 & 0 & \kappa^{j}\\ 0 & b^{j} & 0 & \kappa^{j}\\ 0 & 0 & c^{j} & \kappa^{j} \end{array}\right). \end{array}$$

If point correspondences between images are known, projective reconstruction can be performed by solving a system of quadratic equations.

Quan proposed an algorithm to compute projective invariants of six 3D points from three projection images [9]. Given any six 3D points, the author selected five points as the standard basis as in (2). The six unknown points in 3D space are projective equivalent to the following normalized points:

(5) $$\begin{array}{c} X_{1}=\left(\begin{array}{c} 1\\ 0\\ 0\\ 0 \end{array}\right),\;\;X_{2}=\left(\begin{array}{c} 0\\ 1\\ 0\\ 0 \end{array}\right),\;\;X_{3}=\left(\begin{array}{c} 0\\ 0\\ 1\\ 0 \end{array}\right),\\ X_{4}=\left(\begin{array}{c} 0\\ 0\\ 0\\ 1 \end{array}\right),\;\;X_{5}=\left(\begin{array}{c} 1\\ 1\\ 1\\ 1 \end{array}\right),\;\;X_{6}=\left(\begin{array}{c} X\\ Y\\ Z\\ T \end{array}\right). \end{array}$$

The known point locations in the three 2D images are first normalized according to the projective basis. After this, the known point locations in the *j*th image are then corresponding to

(6) $$\begin{array}{c} x_{1}^{j}=\left(\begin{array}{c} 1\\ 0\\ 0 \end{array}\right),\;\;x_{2}^{j}=\left(\begin{array}{c} 0\\ 1\\ 0 \end{array}\right),\;\;x_{3}^{j}=\left(\begin{array}{c} 0\\ 0\\ 1 \end{array}\right),\\ x_{4}^{j}=\left(\begin{array}{c} 1\\ 1\\ 1 \end{array}\right),\;\;x_{5}^{j}=\left(\begin{array}{c} u_{5}^{j}\\ v_{5}^{j}\\ w_{5}^{j} \end{array}\right),\\ x_{6}^{j}=\left(\begin{array}{c} u_{6}^{j}\\ v_{6}^{j}\\ w_{6}^{j} \end{array}\right),\;j=1,2,3. \end{array}$$

From these correspondence relations, a homogeneous nonlinear equation of the form

(7) i1jXY+i2jXZ+i3jXT+i4jYZ+i5jYT+i6jZT=0

can be derived for the *j*th image, where

(8) $$\begin{array}{c} i_{1}^{j}=w_{6}^{j}\left(u_{5}^{j}-v_{5}^{j}\right),\;\;i_{2}^{j}=v_{6}^{j}\left(w_{5}^{j}-u_{5}^{j}\right),\\ i_{3}^{j}=u_{5}^{j}\left(v_{6}^{j}-w_{6}^{j}\right),\;\;i_{4}^{j}=u_{6}^{j}\left(v_{5}^{j}-w_{5}^{j}\right),\\ i_{5}^{j}=v_{5}^{j}\left(w_{6}^{1}-u_{6}^{j}\right),\;i_{6}^{j}=w_{5}^{j}\left(u_{6}^{j}-v_{6}^{j}\right),\;j=1,2,3. \end{array}$$

It is also noticed that

(9) $$\begin{array}{c} i_{1}^{j}+i_{2}^{j}+i_{3}^{j}+i_{4}^{j}+i_{5}^{j}+i_{6}^{j}=0,\;j=1,2,3. \end{array}$$

Since six 3D points have 18 degrees of freedom and a 3D projective transformation has 15 degrees of freedom, six points in 3D space can have 18 − 15 = 3 independent projective invariants. There are many forms of projective invariants. It is noticed that the ratios of *X*, *Y*, *Z*, and *T* in (7) are projective invariant. The three independent such invariants can be

(10) $$\begin{array}{c} \alpha =\frac{X}{T},\;\;\beta =\frac{Y}{T},\;\;\gamma =\frac{Z}{T}. \end{array}$$

So the goal is to compute these unknown 3D projective invariants from three of the 2D images.

Quan tried to solve the system of bilinear equations (7) using the classical resultant technique. After eliminating the variable *Z*, he obtained two homogeneous polynomial equations of the third degree in three variables

(11) G1≡e11X2Y+e21XY2+e31XYT+e41X2T  +e51XT2+e61Y2T+e61YT2=0,G2≡e12X2Y+e22XY2+e32XYT+e42X2T  +e52XT2+e62Y2T+e62YT2=0.

Eliminating *Y* again will result in a homogeneous polynomial equation in *X* and *T* of degree eight. After that, a third degree polynomial equation can be derived numerically through polynomial factorization of the following form:

(12) XT(X−T)(b1X2+b2XT+b3T2)  ×(a1X3+a2X2T+a3XT2+a4T3)=0.

As we can see from the procedure described above, the method proposed by Quan is hard to implement by ordinary users and inconvenient for real applications. In [13], the author proposed a method to eliminate variable *γ* and variable *β* in a single step. A third degree polynomial equation in single variable *α* was given explicitly.

## 3. A Linear Method to Compute Projective Invariants from 4 Images

A novel direct method for computing projective invariants of six 3D points from four images is presented in this section. We begin by considering a set of 3D points which are seen from four views.

Suppose that a set of six 3D points labeled **X**_*i*_ are given, the geometric structure of which is unknown. The point set is projected into view images by four unknown camera matrices **P** ^1^,  **P** ^2^,  **P** ^3^, and **P** ^4^. The relationships between them are

(13) $$\begin{array}{c} k_{i}^{j}x_{i}^{j}=P^{j}X_{i},\;i=1,\ldots ,6,\;\;j=1,2,3,4. \end{array}$$

The only information available is the point locations in the four images and point correspondences between the four projections

(14) xi1=(ui1vi11)⟷xi2=(ui2vi21)⟷xi3=(ui3vi31)  ⟷xi4=(ui4vi41),

where *i* = 1,…, 6. It is often supposed that no four points in space are coplanar and no three points in the images are collinear. Otherwise the problem is much simpler.

Points **X**_5_ and **X**_6_ can be represented as linear combinations of **X**_1_, **X**_2_, **X**_3_, and **X**_4_

(15) $$\begin{array}{c} X_{5}=\alpha_{1}X_{1}+\alpha_{2}X_{2}+\alpha_{3}X_{3}+\alpha_{4}X_{4},\\ X_{6}=\beta_{1}X_{1}+\beta_{2}X_{2}+\beta_{3}X_{3}+\beta_{4}X_{4}. \end{array}$$

Since points **X**_1_, **X**_2_, **X**_3_, and **X**_4_ are linearly independent, this representation is unique and all the *α*_*i*_ and *β*_*i*_ are nonzero. There are many forms of projective invariants. It is observed that the cross ratios of coefficients in (15) are projective invariant. Six 3D points have 18 degrees of freedom and 3D projective transformation has 15 degrees of freedom. So, six 3D points can have 3 independent projective invariants. A set of functional independent projective invariants of this form are

(16) $$\begin{array}{c} I_{1}=\frac{\alpha_{1}\beta_{2}}{\alpha_{2}\beta_{1}},\;\;I_{2}=\frac{\alpha_{1}\beta_{3}}{\alpha_{3}\beta_{1}},\;\;I_{3}=\frac{\alpha_{1}\beta_{4}}{\alpha_{4}\beta_{1}}.\;\; \end{array}$$

The projective invariance of *I*_1_, *I*_2_, and *I*_3_ can be proved easily. Suppose that the six points **X**_1_, **X**_2_, **X**_3_, **X**_4_, **X**_5_, and **X**_6_ are transformed into **Y**_1_, **Y**_2_, **Y**_3_, **Y**_4_, **Y**_5_, and **Y**_6_ by a 3D projective transformation **A**, where **A** is a 4 × 4 full rank matrix. That is,

(17) $$\begin{array}{c} \lambda_{i}Y_{i}=AX_{i},\;i=1,2,3,4,5,6, \end{array}$$

where *λ*_*i*_ is a nonzero real number. Let

(18) $$\begin{array}{c} Y_{5}={\tilde{\alpha }}_{1}Y_{1}+{\tilde{\alpha }}_{2}Y_{2}+{\tilde{\alpha }}_{3}Y_{3}+{\tilde{\alpha }}_{4}Y_{4},\\ Y_{6}={\tilde{\beta }}_{1}Y_{1}+{\tilde{\beta }}_{2}Y_{2}+{\tilde{\beta }}_{3}Y_{3}+{\tilde{\beta }}_{4}Y_{4} \end{array}$$

be the linear representations of **Y**_5_ and **Y**_6_ in **Y**_1_, **Y**_2_, **Y**_3_, and **Y**_4_. Multiplying each side of each equation in (18) by matrix **A** ^−1^, we have

(19) $$\begin{array}{c} X_{5}=\frac{{\tilde{\alpha }}_{1}}{\lambda_{1}}\lambda_{5}X_{1}+\frac{{\tilde{\alpha }}_{2}}{\lambda_{2}}\lambda_{5}X_{2}+\frac{{\tilde{\alpha }}_{3}}{\lambda_{3}}\lambda_{5}X_{3}+\frac{{\tilde{\alpha }}_{4}}{\lambda_{4}}\lambda_{5}X_{4},\\ X_{6}=\frac{{\tilde{\beta }}_{1}}{\lambda_{1}}\lambda_{6}X_{1}+\frac{{\tilde{\beta }}_{2}}{\lambda_{2}}\lambda_{6}X_{2}+\frac{{\tilde{\beta }}_{3}}{\lambda_{3}}\lambda_{6}X_{3}+\frac{{\tilde{\beta }}_{4}}{\lambda_{4}}\lambda_{6}X_{4}. \end{array}$$

Since vectors **X**_1_, **X**_2_, **X**_3_, and **X**_4_ are linearly independent, the linear representations in (15) and (19) are exactly the same. So we have

(20) $$\begin{array}{c} I_{1}=\frac{\alpha_{1}\beta_{2}}{\alpha_{2}\beta_{1}}=\frac{{\tilde{\alpha }}_{1}{\tilde{\beta }}_{2}}{{\tilde{\alpha }}_{2}{\tilde{\beta }}_{1}}={\tilde{I}}_{1},\\ I_{2}=\frac{\alpha_{1}\beta_{3}}{\alpha_{3}\beta_{1}}=\frac{{\tilde{\alpha }}_{1}{\tilde{\beta }}_{3}}{{\tilde{\alpha }}_{3}{\tilde{\beta }}_{1}}={\tilde{I}}_{2},\\ I_{3}=\frac{\alpha_{1}\beta_{4}}{\alpha_{4}\beta_{1}}=\frac{{\tilde{\alpha }}_{1}{\tilde{\beta }}_{4}}{{\tilde{\alpha }}_{4}{\tilde{\beta }}_{1}}={\tilde{I}}_{3}. \end{array}$$

This proved the invariance of *I*_1_, *I*_2_, and *I*_3_.

The set of projective invariants in (16) have the property that when an invariant equals one, four of the 3D points are coplanar. This can be proved easily. For example, if *I*_1_ = 1, then *α*_1_ *β*_2_ = *α*_2_ *β*_1_. From (15), we have

(21) $$\begin{array}{c} \beta_{1}X_{5}=\alpha_{1}\beta_{1}X_{1}+\alpha_{2}\beta_{1}X_{2}+\alpha_{3}\beta_{1}X_{3}+\alpha_{4}\beta_{1}X_{4},\\ \alpha_{1}X_{6}=\alpha_{1}\beta_{1}X_{1}+\alpha_{1}\beta_{2}X_{2}+\alpha_{1}\beta_{3}X_{3}+\alpha_{1}\beta_{4}X_{4}. \end{array}$$

Subtracting one equation from the other equation in (21), we get

(22) (α1β3−α3β1)X3+(α1β4−α4β1)X4+β1X5−α1X6=0.

Since *α*_1_ and *β*_1_ are not zero, we have a nontrivial linear combination of points **X**_3_, **X**_4_, **X**_5_, and **X**_6_. So they are coplanar.

On the other hand, if points **X**_3_, **X**_4_, **X**_5_, and **X**_6_ are coplanar, then there are numbers *η*_3_,  *η*_4_,  *η*_5_, and  *η*_6_ which are not all zero such that

(23) $$\begin{array}{c} \eta_{3}X_{3}+\eta_{4}X_{4}+\eta_{5}X_{5}+\eta_{6}X_{6}=0. \end{array}$$

Substituting **X**_5_ and **X**_6_ using (15) into (23), we obtain

(24) (α1η5+β1η6)X1+(α2η5+β2η6)X2  +(α3η5+β3η6+η3)X3+(α4η5+β4η6+η4)X4=0.

Since points **X**_1_, **X**_2_, **X**_3_, and **X**_4_ are not coplanar, the coefficients in (24) have to be exactly zero. From this condition we have

(25) $$\begin{array}{c} \alpha_{1}\eta_{5}+\beta_{1}\eta_{6}=0,\;\;\alpha_{2}\eta_{5}+\beta_{2}\eta_{6}=0. \end{array}$$

From (25), we obtain

(26) $$\begin{array}{c} I_{1}=\frac{\alpha_{1}\beta_{2}}{\alpha_{2}\beta_{1}}=1. \end{array}$$

This proved the claim that the necessary and sufficient condition for four of the six points to be coplanar is that one of the projective invariants equals one.

Our next objective is to derive these invariants from image point correspondences. Multiplying each side of (15) by the projection matrices **P** ^1^, **P** ^2^, **P** ^3^, and **P** ^4^, we have

(27) κ5ix5i=α1κ1ix1i+α2κ2ix2i+α3κ3ix3i+α4κ4ix4i,κ6ix6i=β1κ1ix1i+β2κ2ix2i+β3κ3ix3i+β4κ4ix4i,i=1,2,3,4.

That is,

(28) κ5iu5i=α1κ1iu1i+α2κ2iu2i+α3κ3iu3i+α4κ4iu4i,κ5iv5i=α1κ1iv1i+α2κ2iv2i+α3κ3iv3i+α4κ4iv4i,κ5i=α1κ1i+α2κ2i+α3κ3i+α4κ4i,κ6iu6i=β1κ1iu1i+β2κ2iu2i+β3κ3iu3i+β4κ4iu4i,κ6iv6i=β1κ1iv1i+β2κ2iv2i+β3κ3iv3i+β4κ4iv4i,κ6i=β1κ1i+β2κ2i+β3κ3i+β4κ4i,i=1,2,3,4.

Applying variable eliminations to (28), we get

(29) α1κ1ia1i+α2κ2ia2i+α3κ3ia3i+α4κ4ia4i=0,α1κ1ib1i+α2κ2ib2i+α3κ3ib3i+α4κ4ib4i=0,β1κ1ic1i+β2κ2ic2i+β3κ3ic3i+β4κ4ic4i=0,β1κ1id1i+β2κ2id2i+β3κ3id3i+β4κ4id4i=0,i=1,2,3,4,

where

(30) aij=uij−u5j,  bij=vij−v5j,cij=uij−u6j,  dij=vij−v6j,i,j=1,2,3,4.

Rewriting (29) in another form, we have

(31) a1i+a2iK1+a3iK2+a4iK3=0,b1i+b2iK1+b3iK2+b4iK3=0,c1i+c2iI1K1+c3iI2K2+c4iI3K3=0,d1i+d2iI1K1+d3iI2K2+d4iI3K3=0,i=1,2,3,4,

where

(32) $$\begin{array}{c} K_{1}=\frac{\alpha_{2}\kappa_{2}^{1}}{\alpha_{1}\kappa_{1}^{1}},\;\;K_{2}=\frac{\alpha_{3}\kappa_{3}^{1}}{\alpha_{1}\kappa_{1}^{1}},\;\;K_{3}=\frac{\alpha_{4}\kappa_{4}^{1}}{\alpha_{1}\kappa_{1}^{1}}, \end{array}$$

(33) $$\begin{array}{c} I_{1}=\frac{\alpha_{1}\beta_{2}}{\alpha_{2}\beta_{1}},\;\;I_{2}=\frac{\alpha_{1}\beta_{3}}{\alpha_{3}\beta_{1}},\;\;I_{3}=\frac{\alpha_{1}\beta_{4}}{\alpha_{4}\beta_{1}}. \end{array}$$

Since we have known in advance that the systems of equations in (31) have nontrivial solutions, the coefficients matrices in (31) must be rank deficient. That is

(34) det⁡(a1ia2ia3ia4ib1ib2ib3ib4ic1iI1c2iI2c3iI3c4id1iI1d2iI2d3iI3d4i)=0,i=1,2,3,4.

Using these constraints, we can obtain a system of four bilinear equations in variables *I*_1_, *I*_2_, and *I*_3_ of the following form:

(35) t1iI1+t2iI2+t3iI3+t4iI1I2+t5iI1I3+t6iI2I3=0,i=1,2,3,4,

where

(36) t1i=a4ib3ic2id1i−a4ib3ic1id2i+a3ib4ic1id2i−a3ib4ic2id1i,t2i=a4ib2ic1id3i−a4ib2ic3id1i+a2ib4ic3id1i−a2ib4ic1id3i,t3i=a3ib2ic4id1i−a2ib3ic4id1i+a2ib3ic1id4i−a3ib2ic1id4i,t4i=a4ib1ic3id2i−a4ib1ic2id3i+a1ib4ic2id3i−a1ib4ic3id2i,t5i=a1ib3ic4id2i−a3ib1ic4id2i+a3ib1ic2id4i−a1ib3ic2id4i,t6i=a2ib1ic4id3i−a1ib2ic4id3i+a1ib2ic3id4i−a2ib1ic3id4i,i=1,2,3,4.

The system of bilinear equations in (35) can be solved directly by numerical methods. However, nonlinear numerical methods are usually time consuming and sometimes not very stable. Classical method of variable elimination through the resultant technique will result in a high order polynomial equation in a single variable. This is not what we anticipate. The main contribution of this paper is that we will show that the system of nonlinear equations can be solved linearly. This is done by using a modified scheme of variable elimination.

Now we proceed to derive the linear solution of the system of equations (35). Rewriting (35) in matrix form, we can obtain

(37) $$\begin{array}{c} \left(\begin{array}{cccc} t_{1}^{1} & t_{2}^{1}+t_{4}^{1}I_{1} & t_{3}^{1}+t_{5}^{1}I_{1} & t_{6}^{1}\\ t_{1}^{2} & t_{2}^{2}+t_{4}^{2}I_{1} & t_{3}^{2}+t_{5}^{2}I_{1} & t_{6}^{2}\\ t_{1}^{3} & t_{2}^{3}+t_{4}^{3}I_{1} & t_{3}^{3}+t_{5}^{3}I_{1} & t_{6}^{3}\\ t_{1}^{4} & t_{2}^{4}+t_{4}^{4}I_{1} & t_{3}^{4}+t_{5}^{4}I_{1} & t_{6}^{4} \end{array}\right)\left(\begin{array}{c} I_{1}\\ I_{2}\\ I_{3}\\ I_{2}I_{3} \end{array}\right)=0. \end{array}$$

Since *I*_1_, *I*_2_, and *I*_3_ are nonzero, the determinant of the coefficient matrix in (37) has to be zero. So we have

(38) $$\begin{array}{c} \det\left(\begin{array}{cccc} t_{1}^{1} & t_{2}^{1}+t_{4}^{1}I_{1} & t_{3}^{1}+t_{5}^{1}I_{1} & t_{6}^{1}\\ t_{1}^{2} & t_{2}^{2}+t_{4}^{2}I_{1} & t_{3}^{2}+t_{5}^{2}I_{1} & t_{6}^{2}\\ t_{1}^{3} & t_{2}^{3}+t_{4}^{3}I_{1} & t_{3}^{3}+t_{5}^{3}I_{1} & t_{6}^{3}\\ t_{1}^{4} & t_{2}^{4}+t_{4}^{4}I_{1} & t_{3}^{4}+t_{5}^{4}I_{1} & t_{6}^{4} \end{array}\right)=0. \end{array}$$

This is a second degree polynomial equation in variable *I*_1_. A quadratic equation generally has two solutions. To obtain a unique solution, we have to apply further constraints. It is checked that

(39) t1i+t2i+t3i+t4i+t5i+t6i=0, i=1,2,3,4.

Applying constraints (39) to (38), we obtain the following equation:

(40) $$\begin{array}{c} \det\left(\begin{array}{cccc} t_{1}^{1} & \left(t_{4}^{1}+t_{5}^{1}\right)\left(I_{1}-1\right) & t_{3}^{1}+t_{5}^{1}I_{1} & t_{6}^{1}\\ t_{1}^{2} & \left(t_{4}^{2}+t_{5}^{2}\right)\left(I_{1}-1\right) & t_{3}^{2}+t_{5}^{2}I_{1} & t_{6}^{2}\\ t_{1}^{3} & \left(t_{4}^{3}+t_{5}^{3}\right)\left(I_{1}-1\right) & t_{3}^{3}+t_{5}^{3}I_{1} & t_{6}^{3}\\ t_{1}^{4} & \left(t_{4}^{4}+t_{5}^{4}\right)\left(I_{1}-1\right) & t_{3}^{4}+t_{5}^{4}I_{1} & t_{6}^{4} \end{array}\right)=0. \end{array}$$

The solutions of (40) are *I*_1_ = 1 and

(41) $$\begin{array}{c} I_{1}=-\frac{\det\left(\begin{array}{cccc} t_{1}^{1} & t_{4}^{1}+t_{5}^{1} & t_{3}^{1} & t_{6}^{1}\\ t_{1}^{2} & t_{4}^{2}+t_{5}^{2} & t_{3}^{2} & t_{6}^{2}\\ t_{1}^{3} & t_{4}^{3}+t_{5}^{3} & t_{3}^{3} & t_{6}^{3}\\ t_{1}^{4} & t_{4}^{4}+t_{5}^{4} & t_{3}^{4} & t_{6}^{4} \end{array}\right)}{\det\left(\begin{array}{cccc} t_{1}^{1} & t_{4}^{1} & t_{5}^{1} & t_{6}^{1}\\ t_{1}^{2} & t_{4}^{2} & t_{5}^{2} & t_{6}^{2}\\ t_{1}^{3} & t_{4}^{3} & t_{5}^{3} & t_{6}^{3}\\ t_{1}^{4} & t_{4}^{4} & t_{5}^{4} & t_{6}^{4} \end{array}\right)}. \end{array}$$

The solution *I*_1_ = 1 corresponds to the condition that four of the 3D points are coplanar. We neglect this solution according to the assumption that no four points are coplanar. In this way a unique linear solution of the projective invariant *I*_1_ is obtained.

Now we derive the solution of *I*_2_. From (35), we can obtain

(42) $$\begin{array}{c} \left(\begin{array}{cccc} t_{1}^{1}+t_{4}^{1}I_{2} & t_{2}^{1} & t_{3}^{1}+t_{6}^{1}I_{2} & t_{5}^{1}\\ t_{1}^{2}+t_{4}^{2}I_{2} & t_{2}^{2} & t_{3}^{2}+t_{6}^{2}I_{2} & t_{5}^{2}\\ t_{1}^{3}+t_{4}^{3}I_{2} & t_{2}^{3} & t_{3}^{3}+t_{6}^{3}I_{2} & t_{5}^{3}\\ t_{1}^{4}+t_{4}^{4}I_{2} & t_{2}^{4} & t_{3}^{4}+t_{6}^{4}I_{2} & t_{5}^{4} \end{array}\right)\left(\begin{array}{c} I_{1}\\ I_{2}\\ I_{3}\\ I_{1}I_{3} \end{array}\right)=0. \end{array}$$

Since *I*_1_, *I*_2_, and *I*_3_ are nonzero, we have

(43) $$\begin{array}{c} \det\left(\begin{array}{cccc} t_{1}^{1}+t_{4}^{1}I_{2} & t_{2}^{1} & t_{3}^{1}+t_{6}^{1}I_{2} & t_{5}^{1}\\ t_{1}^{2}+t_{4}^{2}I_{2} & t_{2}^{2} & t_{3}^{2}+t_{6}^{2}I_{2} & t_{5}^{2}\\ t_{1}^{3}+t_{4}^{3}I_{2} & t_{2}^{3} & t_{3}^{3}+t_{6}^{3}I_{2} & t_{5}^{3}\\ t_{1}^{4}+t_{4}^{4}I_{2} & t_{2}^{4} & t_{3}^{4}+t_{6}^{4}I_{2} & t_{5}^{4} \end{array}\right)=0. \end{array}$$

Applying constraints (39) to (43), we obtain

(44) $$\begin{array}{c} \det\left(\begin{array}{cccc} \left(t_{4}^{1}+t_{6}^{1}\right)\left(I_{2}-1\right) & t_{2}^{1} & t_{3}^{1}+t_{6}^{1}I_{2} & t_{5}^{1}\\ \left(t_{4}^{2}+t_{6}^{2}\right)\left(I_{2}-1\right) & t_{2}^{2} & t_{3}^{2}+t_{6}^{2}I_{2} & t_{5}^{2}\\ \left(t_{4}^{3}+t_{6}^{3}\right)\left(I_{2}-1\right) & t_{2}^{3} & t_{3}^{3}+t_{6}^{3}I_{2} & t_{5}^{3}\\ \left(t_{4}^{4}+t_{6}^{4}\right)\left(I_{2}-1\right) & t_{2}^{4} & t_{3}^{4}+t_{6}^{4}I_{2} & t_{5}^{4} \end{array}\right). \end{array}$$

Then the unique solution of *I*_2_ is

(45) $$\begin{array}{c} I_{2}=-\frac{\det\left(\begin{array}{cccc} t_{4}^{1}+t_{6}^{1} & t_{2}^{1} & t_{3}^{1} & t_{5}^{1}\\ t_{4}^{2}+t_{6}^{2} & t_{2}^{2} & t_{3}^{2} & t_{5}^{2}\\ t_{4}^{3}+t_{6}^{3} & t_{2}^{3} & t_{3}^{3} & t_{5}^{3}\\ t_{4}^{4}+t_{6}^{4} & t_{2}^{4} & t_{3}^{4} & t_{5}^{4} \end{array}\right)}{\det\left(\begin{array}{cccc} t_{4}^{1} & t_{2}^{1} & t_{6}^{1} & t_{5}^{1}\\ t_{4}^{2} & t_{2}^{2} & t_{6}^{2} & t_{5}^{2}\\ t_{4}^{3} & t_{2}^{3} & t_{6}^{3} & t_{5}^{3}\\ t_{4}^{4} & t_{2}^{4} & t_{6}^{4} & t_{5}^{4} \end{array}\right)}. \end{array}$$

Now we derive the solution of *I*_3_. From (35), we can obtain

(46) $$\begin{array}{c} \left(\begin{array}{cccc} t_{1}^{1}+t_{5}^{1}I_{3} & t_{2}^{1}+t_{6}^{1}I_{3} & t_{3}^{1} & t_{4}^{1}\\ t_{1}^{2}+t_{5}^{2}I_{3} & t_{2}^{2}+t_{6}^{2}I_{3} & t_{3}^{2} & t_{4}^{2}\\ t_{1}^{3}+t_{5}^{3}I_{3} & t_{2}^{3}+t_{6}^{3}I_{3} & t_{3}^{3} & t_{4}^{3}\\ t_{1}^{4}+t_{5}^{4}I_{3} & t_{2}^{4}+t_{6}^{4}I_{3} & t_{3}^{4} & t_{4}^{4} \end{array}\right)\left(\begin{array}{c} I_{1}\\ I_{2}\\ I_{3}\\ I_{1}I_{2} \end{array}\right)=0. \end{array}$$

Since *I*_1_, *I*_2_, and *I*_3_ are nonzero, we have

(47) $$\begin{array}{c} \det\left(\begin{array}{cccc} t_{1}^{1}+t_{5}^{1}I_{3} & t_{2}^{1}+t_{6}^{1}I_{3} & t_{3}^{1} & t_{4}^{1}\\ t_{1}^{2}+t_{5}^{2}I_{3} & t_{2}^{2}+t_{6}^{2}I_{3} & t_{3}^{2} & t_{4}^{2}\\ t_{1}^{3}+t_{5}^{3}I_{3} & t_{2}^{3}+t_{6}^{3}I_{3} & t_{3}^{3} & t_{4}^{3}\\ t_{1}^{4}+t_{5}^{4}I_{3} & t_{2}^{4}+t_{6}^{4}I_{3} & t_{3}^{4} & t_{4}^{4} \end{array}\right)=0. \end{array}$$

Applying constraints (39) to (47), we obtain

(48) $$\begin{array}{c} \det\left(\begin{array}{cccc} \left(t_{5}^{1}+t_{6}^{1}\right)\left(I_{3}-1\right) & t_{2}^{1}+t_{6}^{1}I_{3} & t_{3}^{1} & t_{4}^{1}\\ \left(t_{5}^{2}+t_{6}^{2}\right)\left(I_{3}-1\right) & t_{2}^{2}+t_{6}^{2}I_{3} & t_{3}^{2} & t_{4}^{2}\\ \left(t_{5}^{3}+t_{6}^{3}\right)\left(I_{3}-1\right) & t_{2}^{3}+t_{6}^{3}I_{3} & t_{3}^{3} & t_{4}^{3}\\ \left(t_{5}^{4}+t_{6}^{4}\right)\left(I_{3}-1\right) & t_{2}^{4}+t_{6}^{4}I_{3} & t_{3}^{4} & t_{4}^{4} \end{array}\right)=0. \end{array}$$

Then the unique solution of *I*_3_ is

(49) $$\begin{array}{c} I_{3}=-\frac{\det\left(\begin{array}{cccc} t_{5}^{1}+t_{6}^{1} & t_{2}^{1} & t_{3}^{1} & t_{4}^{1}\\ t_{5}^{2}+t_{6}^{2} & t_{2}^{2} & t_{3}^{2} & t_{4}^{2}\\ t_{5}^{3}+t_{6}^{3} & t_{2}^{3} & t_{3}^{3} & t_{4}^{3}\\ t_{5}^{4}+t_{6}^{4} & t_{2}^{4} & t_{3}^{4} & t_{4}^{4} \end{array}\right)}{\det\left(\begin{array}{cccc} t_{5}^{1} & t_{6}^{1} & t_{3}^{1} & t_{4}^{1}\\ t_{5}^{2} & t_{6}^{2} & t_{3}^{2} & t_{4}^{2}\\ t_{5}^{3} & t_{6}^{3} & t_{3}^{3} & t_{4}^{3}\\ t_{5}^{4} & t_{6}^{4} & t_{3}^{4} & t_{4}^{4} \end{array}\right)}. \end{array}$$

## 4. Implementation of the Algorithm

We have validated the proposed method on the *mathematica* platform. The implementation is very simple. The code is given in Algorithm 1.

## 5. Conclusions

We have presented a direct and linear method for computing projective invariants of six 3D points from four 3D to 2D projection images. It can be used in 3D point pattern recognition from 2D images directly. Traditional methods for solving this problem are nonlinear and very complicated to use in real applications. The proposed formulas are clear and easy to implement by ordinary users. Another feature of our method is that we compute the projective invariants using only the original data. It is noticed that transformations of the original data can amplify the noise level of the data. This study provides a deeper understanding of the structure and motion problem. It seems that the natural configuration of the projective reconstruction problem is six points and four images.

Future directions of research include using this method in iterative or minimization schemas to solve the projective reconstruction problem with noising data, missing data, or outliers. It is also possible to develop similar methods for the cases of seven points in three images and eight points in two images.

## Figures and Tables

**Algorithm 1:**